# Methodological Critique of Littman’s (2018) Parental-Respondents Accounts of “Rapid-Onset Gender Dysphoria”

**DOI:** 10.1007/s10508-019-1453-2

**Published:** 2019-04-22

**Authors:** Arjee Javellana Restar

**Affiliations:** Department of Behavioral and Social Sciences, Brown University School of Public Health, 121 South Main Street, Box G-S 121-2, Providence, RI 02903 USA

## Introduction

Littman’s (2018) descriptive paper reports on parents’ observations (i.e., parental-respondents accounts) of adolescent and young adults with “rapid-onset gender dysphoria” (ROGD), an allegedly new type of gender dysphoria that is not listed in the *Diagnostic and Statistical Manual of Mental Disorders* (DSM-5) (American Psychiatric Association, 2013). Using an online quantitative and qualitative survey that sampled parents who frequented three distinct Web sites, the research aims were to: (1) describe the presentation of “ROGD” (defined as the presentations of transgender (trans)-identified adolescents and young adults who did not appear to meet clinical criteria for gender dysphoria during childhood, yet suddenly exhibit gender dysphoria during or after puberty by disclosing their trans identities to their parents) and (2) generate hypotheses based on the premise that “social and peer contagion” is a key determinant of “ROGD.”

### Framework and Premise

Principles of research methods necessitate that a study’s design must be appropriate to the aims of the study and the context of the phenomenon (Cozby, 2012; Ruane, 2005). In Littman’s case, the majority of methodological and design issues stem from the use of a pathologizing framework and language of pathology to conceive, describe, and theorize the phenomenon as tantamount to both an infectious disease (“cluster outbreaks of gender dysphoria”) and a disorder (e.g., “eating disorders and anorexia nervosa”) (Littman, 2018). Consequentially, the study design and interpretation of the results are framed with this pathology framework. Specifically, the article begins with the premise of conceptualizing gender dysphoria and trans identification as partly a consequence of “social and peer contagion” that “spreads of affect or behaviors through a population… [and] where an individual and peer mutually influence each other in a way that promotes emotions and behaviors that can potentially undermine their own development or harm others” (Littman, 2018). The article continues to pathologize gender dysphoria and affirmation of trans identification through social network peers and online environments as an example of “deviancy training,” and describing it as an unhealthy pattern of reinforcement with trans-identified peers and linking it with a behavior that is “deceiving parents and doctors” (Littman, 2018). The pathologizing lens used by Littman to study this gender dysphoria-related phenomenon speaks to the researcher’s a priori bias that is manifested in the construction of measurements and methodologies deliberately chosen to investigate this phenomenon.

Identifying as transgender is not a disease nor is it considered a mental disorder by the American Psychiatric Association (2013), the World Professional Association for Transgender Health (WPATH, 2011), and the World Health Organization (2018). While distress associated with gender dysphoria is diagnosable, this diagnosis is not to be used for stigmatization or pathologization (Coleman et al., 2012). The American Psychiatric Association (2013) explicitly states that the DSM-5 “aims to avoid stigma.” As such, it is vital that the DSM is to be utilized with this spirit and mission. Choosing to use a specific framework and language that continues to pathologize trans youths and young adults is the exact opposite and reflects a certain preexisting non-neutrality bias of studying the phenomenon. For any researcher studying to improve the lives of trans-identified youths and young adults (including their parents), it is vital to note and to acknowledge the body of validated work that has been and continue to be built into understanding transgender health, including etiology of gender dysphoria, and to use methodologies and frameworks that are not furthering the pathologization and stigmatization of this historically vulnerable and marginalized population.

### Consent

The premise of the study is also stated at the beginning of the consent form (publically available as “supporting information” with the *PLoS One* article) and introduces risk for participant’s self-selection bias and survey response bias. Self-selection is a type of bias that, when introduced in the survey, motivates participants (i.e., parental-respondents) to elect themselves into the study such that this selection is different from the study enrollment criteria (Bethlehem, 2010; Cozby, 2012; Delgado-Rodriguez & Llorca, 2004; Ruane, 2005). Littman (2018) describes the “social and peer contagion” premise extensively at the beginning of the consent document: This phenomenon is “in the context of increased social media/Internet use and/or being part of a peer group in which one or multiple friends have developed gender dysphoria and come out as transgender during a similar time frame. Several parents have described situations where entire friend groups became gender dysphoric.” This “social and peer contagion” premise is not a part of the enrollment criteria. Providing this premise prior during the consent process provides an opportunity for motivating a specific group of parental-respondents, particularly those who agree with the premise, to elect to participate in the survey. Furthermore, providing the premise of the study in this way sets expectations of the survey before parental-respondents can even begin to provide their answers, which can bias their response toward support for the premise.

### Enrollment

As criteria for enrollment, Littman (2018) asked parents to indicate based on their observation if their adolescent child has “ROGD” and whether it started during or after puberty. Littman also provided definitions for “gender dysphoria,” “transgender,” and “coming out/announcing as transgender,” but not specifically “ROGD” and “puberty.” It is unclear whether parents were informed how “ROGD” and puberty were operationally defined and conceptualized in this paper. Specifically, what makes gender dysphoria a sudden or rapid phenomenon solely based on parents’ accounts of adolescents and young adults’ announcement of their trans identities? As definitions for puberty have been contested in the past and recently (Coleman & Coleman, 2002; Dorn, Dahl, Woodward, & Biro, 2006; Kaplowitz & Oberfield, 1999; Sawyer, Azzopardi, Wickremarathne, & Patton, 2018), it is unknown what puberty means from the perspective of parents, and whether there were knowledge-based differences that can distort findings based on the operationalization of these terms (Lippold, Coffman, & Greenberg, 2014).

Littman (2018) also asked parents to perform two independent “diagnoses” of their child’s gender dysphoria using the DSM-5 criteria for gender dysphoria in (1) childhood and (2) in adolescence and adulthood (i.e., current age) (American Psychiatric Association, 2013); Littman also noted that the language for these measurements was simplified or adapted for parents. Littman neither provided examples of this simplified version of the DSM-5 nor offered evidence about whether best-practice methods for measure adaptation were used prior to administering the survey. These established methods include but are not limited to cognitive interviewing, confirmatory factor analysis, reliability and procedural validity, and diagnostic criterion validity; each of these methods enhances the likelihood that a newly adapted version of a diagnostic measure retains its original construct and validity (Benson & Clark, 1982; Ruane, 2005; Thompson, 2004; Willis, 2004). Without methodologically confirming the new versions of these two independent diagnostic criteria prior to administration of the survey, instrument bias may have been introduced.

In addition to using non-validated adapted versions of the DSM-5 measurement, another fundamental methodological error Littman (2018) makes is using parental-respondents accounts of “ROGD” to generate interpretations and conclusions about clinical conditions like gender dysphoria. Part of the DSM-5’s diagnostic measurement for gender dysphoria also requires an evaluation of its association with clinically significant distress (American Psychiatric Association, 2013). Unless parents in this paper received formal training and have licenses to conduct clinical psychiatric diagnoses, parents enrolled were not qualified to classify any persons, including their children’s gender dysphoria. Gender dysphoria involves a formalized evaluation that has physical and psychological components, both of which are not easily observable unless one is formally trained (Coleman et al., 2012). As such, relying on parental-respondents’ accounts introduces a significant bias that affects their ability to “diagnose.” It has been previously suggested that parents are less capable of conceptualizing and interpreting their children’s emotional and physical experiences in a manner that is conducive to an observational report (Davis et al., 2007), such as an online survey.

Reliance on retrospective reports is another reason for why parental-respondents accounts of “ROGD” is methodologically inappropriate for examining this phenomenon (Hardt & Rutter, 2004). Littman (2018) asked parents to recall their children’s behavior both in childhood and in their current age. On average, there were at least 6 years for parents to remember between their child’s “childhood” and current age. Asking parents to recollect information on this time frame places a substantial burden on memory (Hassan, 2006). Additionally, while studies on gender identity have contested the validity of retrospective accounts of participants’ own recollection in the past (Bailey & Zucker, 1995), Littman’s methods did not ask trans youth’s own recollection in regard to their own experiences; rather, these recollections were a derivation from their parents. While developmental research has utilized recall methods in the past (Dex, 1995; Hardt & Rutter, 2004), the paper did not provide information on whether there were any tests performed to examine the accuracy of the recall methods. Placing substantial burden on parents’ memory as well as deriving trans youth’s experiences generate increased fallibility, recall bias, and misclassification of “ROGD.”

### Selection and Sampling

Also of concern is the demographic profile of the parental-respondents in this paper. The parental-respondents displayed very narrow demographic stratification despite being sampled from a very specific venue: 82.8% were female sex at birth, 91.4% were White, 99.2% were non-Hispanic, 66.1% were aged 46–60, and 70.9% had attended college. Notably, 76.5% believed that their child’s trans identification is not correct, and recruitment relied heavily on three particular Web sites known to be frequented by parents specifically voicing out and promoting the concept of “ROGD.” Thus, these are not just “worried parents,” but rather a sample of predominantly White mothers who have strong oppositional beliefs about their children’s trans identification and who harbor suspicions about their children having “ROGD.” Furthermore, this non-heterogenous sample of parental-respondents already have “buy-in” about the concept of “ROGD” by frequenting three distinct Web sites known for telling parents not to believe their child is transgender. There is very little evidence that this sample is representative of the diverse parents of trans youths and young adults.

While descriptive studies frequently use convenience sampling, there is a clear distinction between convenience sampling and biased sampling that is not acknowledged by Littman (2018). Participants recruited into a study should never be selected based on a researcher’s a priori knowledge of how the results of the paper would appear and confirm their premise (Cohen & Crabtree, 2006; Grimes & Schulz, 2002). As noted earlier, Littman recruited specifically on three Web sites solely because these venues are attracting a specific demographic group of parental-respondents who are already subscribed into, are selecting into (i.e., self-selection bias), are promoting the concept of “ROGD,” and agree via consent form with the premise of the study. By choosing a specific population of interest and selecting cases and venues where cases can be found, an a priori motivation that favors the investigator’s premise and specific perspectives is likely to be gathered from the sample and thus likely contributing to systemically biased results.

In addition, as the survey was administered online, Littman made no mention of best-practice strategies for conducting web-based surveys (Eysenbach, 2004; Umbach, 2004; Wright, 2005). For example, there was the lack of description of online security against robots and/or Internet “trolls,” including those who are repeat testers, which are known to happen in online studies (Eysenbach, 2004; Wright, 2005). There was no description in the article that conveys the survey had a de-duplication protocol that flags possible multiple responses from the same parental-respondent (i.e., matching IP addresses, assignment of unique “cookies,” or having a feature that disallows the survey to be taken more than once from the same device). Therefore, it is plausible that these data may contain multiple responses from the same parental-respondent. In fact, as evident in the consent document, Littman (2018) decided not to collect IP addresses and explicitly stated that multiple responses from the same parental-respondent who reported having more than one child they suspect to have “ROGD” were allowed by “using one survey to describe one child, a second survey to describe a second child, etc.” Littman did not provide any evidence for controlling or weighting for multiple children from the same family in the analysis and failed to report whether any parental-respondents did indeed have multiple children they observed to have “ROGD.”

### Measurements

Another methodological and analytical error Littman makes in this paper is in the lack of evidence for reliability and internal validity of measures. The study used a descriptive quantitative–qualitative design based on a 90-question survey created by Littman. As the study provided no psychometric information on the survey items or any suggestion that such analysis was conducted once data were gathered, uncertainty arises about the reliability and internal validity of the data and confidence in the results (Benson & Clark, 1982; Ruane, 2005; Thompson, 2004). With the exception of incorporating the adapted DSM-related measures, which as noted above can be questioned regarding the nature of adaptation, Littman (2018) made no references or citations to other valid instruments and problematically used non-validated measures throughout the paper to support the study premise and hypotheses. For example, as Littman (2018) was interested in coping emotions, for which there are an already established battery of validated measurements available (Sveinbjornsdottir & Thorsteinsson, 2008), it is questionable that Littman chose to craft survey questions without any statistical psychometric validation instead of using or adapting validated coping measures.

In the same vein, Littman’s (2018) creation of survey items about coping emotions to support the study hypothesis that “ROGD” was maladaptive is inadequately constructed. For example, Littman asked parents how their child handled strong emotions, with the following response options: “(1) My child is overwhelmed by strong emotions and goes to great lengths to avoid feeling them; (2) my child is overwhelmed by strong emotions and tries to avoid feeling them; (3) my child neither avoids nor seeks out strong emotions; (4) my child tries to seek out situations in order to feel strong emotions; and (5) my child goes to great lengths to seek out situations in order to feel strong emotions.” These response choices are inconsistent to the principles of Likert scale techniques (Likert, 1932) and do not follow essential aspects of directionality (i.e., positive–negative) and intensity (i.e., the strength of emotion). Littman does not provide a definition of what “strong emotions” mean and how this concept is operationalized. Additionally, Littman also collapsed the first two responses in analysis without providing a specific rationale. As there are no indications of pretesting and/or validating these measurements prior to administration, it is unclear whether parental-respondents understood these types of questions and how they responded to them. Lastly, the creation of di-/tri-chotomization variables based on continuous variables throughout the analyses without providing justifications or references from the literature is methodologically unconvincing and reflects a potential for selective coding of negative coping and poor emotional control in support the hypothesis.

In order to counteract interpretation that parental-respondents held general transphobic or heterosexist attitudes, Littman (2018) reported that 85.9% were in favor of “allowing gay and lesbian couples to marry legally” and 88.2% believed that “transgender people deserve the same rights and protections as others.” However, these were only two of four gender and sexual minority-related attitude items included in the survey. It is unclear why these two specific items were selectively reported. The two items not reported concerned parental beliefs around whether it is a good or bad thing (or neither) in society that more gay and lesbian couples are raising children, and whether parental-respondents would support or oppose a law to protect transgender people from discrimination in employment and housing. Both items provide additional depth into the respondents’ attitudes toward gender and sexual minorities, including transgender people. It is unclear why these items were not reported, raising questions about potential reporting bias in which only those items that support the assertion that parental-respondents did not hold negative attitudes against trans people (Cozby, 2012; Ruane, 2005).

### Analyses

A statistical issue of concern that arises from the lack of reliable and internally valid measures is Littman’s (2018) incorrect statistical conclusion validity or the validity of inferences made about the relationship between two variables (Shadish, Cook, & Campbell, 2002). In particular, using the inappropriate deduction of the association between increased exposure to social influence (i.e., friend groups and social media/Internet content) and parental-respondents accounts of “ROGD,” to support the “social and peer contagion” premise without controlling for differences in friend groups, social media, and Internet content is problematic. For the past decade, there is both a worldwide and U.S. trend of increasing screen time and use across all age groups (Pew Research Center, 2010, 2018). Moreover, the justification for using non-sophisticated analytical software to understand the phenomenon through univariate and bivariate analyses falls short, making it unclear whether the analytic exploration directly speaks to the parameters of the research aims. As such, this statistical issue undermines the extent to which any support for the “social and peer contagion” hypothesis can be drawn for all subsequent analyses conducted examining the relationships between the social influence construct and other variables.

Due to biases noted previously, the quality and validity of the findings are critically undermined. For example, Littman (2018) found that a small portion of the parents sampled (23.8%) reported that “[their] child was offered prescriptions for puberty blockers and/or cross-sex hormones at the first visit.” This finding is alarming given that it runs contrary to the WPATH’s Standards of Care for assessing and referring patients for hormone therapy (WPATH, 2011), and, if true, needs clinical attention, as well as in contrast with the current literature. Some studies that sampled transgender patients have documented difficultness of accessing hormones and care due to multiple barriers, including limited and delayed access to pubertal blockers and cross-sex hormones (Gridley et al., 2016; Roberts & Fantz, 2014), and lack of access (Safer et al., 2016). Other studies that have sampled health care providers of transgender youths have found that providers are substantially less comfortable or reluctant in prescribing hormonal care (Poteat, German, & Kerrigan, 2013; Stieglitz, 2010; Thomas & Safer, 2015; Vance, Halpern-Felsher, & Rosenthal, 2015). As such, it is important to elucidate the results of the Littman (2018) paper in light of its methodological limitations as well as alongside the body of transgender health literature.

With regard to the qualitative component of the study design and analysis, Littman (2018) states the use of a “grounded theory approach” to analyze open-ended responses “because it allowed the researcher to assemble the data in accordance with the salient points the respondents were making without forcing the data into a preconceived theoretical framework of the researcher’s own choosing.” The a priori biases present in Littman’s framing of the study and methodological biases identified in the sampling approach, informed consent language, and item selection violate the essential principles of grounded theory (Glaser & Strauss, 2017). A hallmark and a necessary research process of grounded theory is inductive analysis of data (rather than deductive theory-driven analysis) in order to formulate hypotheses (Breckenridge & Jones, 2009; Butler, Copnell, & Hall, 2018; Charmaz, 2014; Glaser & Strauss, 2017), which does not characterize Littman’s paper due to the biases that shaped the study design, sampling, recruitment, and survey construction.

Lastly, Littman (2018) only examined two questions for “full qualitative analysis of themes (one question on friend group behaviors and one on clinician interactions).” Similar to reporting some quantitative measures but not others (e.g., gender and sexual minority-related attitude items), there was no clear rationale provided why only two questions were selectively chosen to be fully analyzed for qualitative purposes.

## Conclusion

A common error in reports of descriptive studies is overstepping the study design and data (Grimes & Schulz, 2002) and Littman’s (2018) paper, for reasons described in this critique, is an example of this fundamental error. Littman’s methodological flaws in the conceptualization and design of the study illustrate the importance of and need for more rigorous survey design and data analysis in descriptive studies. In the context of research with transgender people, who have historically been subjected to pathologizing research, flawed methodologies that lead to tenuous conclusions can have serious implications. While most science has limitations, researchers studying in the field of transgender health should strive to design their studies with appropriate, non-stigmatizing, and non-pathologizing research aims and methods that are grounded in the lived perspectives and experiences of transgender populations.
